# Exploring the Genetic Basis of Wild Boar (*Sus scrofa*) and Its Connection to Classical Swine Fever Spread

**DOI:** 10.1155/tbed/9881511

**Published:** 2025-05-07

**Authors:** Rie Saito, Natsuko Ito Kondo, Yui Nemoto, Toshimasa Takeda, Kosuke Kanda, Nobuyoshi Nakajima, James C. Beasley, Masanori Tamaoki

**Affiliations:** ^1^Department of Animal Science, Faculty of Agriculture, Iwate University, 18-8 Ueda 3-chome, Morioka, Japan; ^2^Savannah River Ecology Laboratory, University of Georgia, P.O. Drawer E, Aiken, South Carolina 29802, USA; ^3^Fukushima Prefectural Centre for Environmental Creation, 10-2 Fukasaku, Miharu-machi 963-7700, Japan; ^4^Biodiversity Division, National Institute for Environmental Studies, 16-2 Onogawa, Tsukuba 305-8506, Japan; ^5^Okutama Practice Forest, Tokyo University of Agriculture, Hikawa 2137, Okutama 198-0212, Japan; ^6^Fukushima Regional Collaborative Research Center, National Institute for Environmental Studies, 10-2 Fukasaku, Miharu-machi 963-7700, Japan; ^7^Warnell School of Forestry and Natural Resources, University of Georgia, 180 East Green St, Athens, Georgia, USA

## Abstract

Classical swine fever (CSF) is the one of the most devastating contagious diseases in domestic swine and wild boar/pigs (*Sus scrofa*). Population genetics is often used to estimate animal dispersal and can also help evaluate host population connectivity, which is crucial for understanding pathogen dispersal. We surveyed genetic population structure of boars using MIG-seq analysis to clarify the geographic barriers that influence boar dispersal in north-central Japan and to demonstrate the relationship between the spread of CSF infection among boars and their population structure. We obtained 382 single-nucleotide polymorphisms from 348 wild boar samples, and the results of STRUCTURE analysis indicated that the highest Δ*K* value was at *K* = 2, followed by *K* = 4. Based on these results, it is evident that the Abukuma river, a major river within north-central Japan, does not act as a barrier to the gene flow of boars, but rather that human infrastructure hinders their dispersal. Further, according to the time series change in the capture site of CSF-infected wild boar and the sum of the probability of belonging to each of the four clades in individual CSF-infected wild boar, our results indicated that the genetic structure of boar populations was correlated with the outbreak pathway of CSF across our study region. Our study suggests that predictions of disease spread, especially for widely distributed host species, is challenging because of the risk of cryptic breaks and changes in wide range connectivity; however, understanding the genetic population structure of wild boar can be a useful tool for predicting the spread of CSF. We concluded that genetic analysis of host population structure may have the possibility to improve predictions of the future dynamics of disease spread.

## 1. Introduction

In today's globalized world, the international movement and transfer of people, livestock, and wildlife poses an increased risk for the introduction and spread of many diseases to human society and wildlife. Classical swine fever (CSF) is the one of the most devastating contagious diseases in domestic swine and wild boar and wild pigs (*Sus scrofa*) [1, 2]. CSF was prevalent in Europe and America by the 1860s, and it is still widespread globally in Asia, Europe, Central and South America, and parts of Africa, although Australia, New Zealand, and North America are currently free of the disease [3, 4]. CSF is a significant concern affecting pig production in Asia, which is a leading market in the global pork supply [5]. In the case of Japan, following eradication efforts in 2015 was officially recognized as CSF-free by the World Organization for Animal Health. However, CSF infection was found in both domestic pig farms and wild boar in Gifu prefecture, located in the central part of Japan, in August 2018 [2, 6]. The CSF virus was closely related to CSFV-2.1, which is detected in East Asia [7, 8]. In addition, according to epidemiological research, domestic pigs in Gifu Prefecture were infected with CSF by wild boars that had already been infected in Japan [7, 8]. CSF exhibits high virulence in domestic pigs; consequently, once a CSF outbreak occurs, it results in the restriction of export markets and necessitates the forced large-scale culling of domestic stock [9]. CSF also heavily impacts wild boar populations, and in regions with wild boar/pigs, transfer between wild boar/pigs and domestic pigs can occur, prompting intensive monitoring and management programs in many regions.

Transmission of the CSF virus (hereafter, CSFV) is caused by direct contact with infected animals, ingestion of contaminated food, and other indirect routes of transmission, such as vehicles, persons, equipment, wild birds, or small animals [10]. Once introduced, CSF can spread rapidly within a population. To decrease the proportion of susceptible animals in a population, oral mass vaccination (OMV) has been implemented in wild boar to prevent the spread of CSFV [1]. According to the European Food Safety Authority (EFSA), OMV baits have been delivered under the soil surface to avoid consumption by non-target species and to maintain baits at fresh temperatures. In addition, aircraft have also been used to deliver vaccine baits, but are less commonly used because of high costs [1]. Such vaccination efforts are time intensive and costly, and thus development of optimized strategies for delivering vaccine baits is of high importance.

Population genetics are useful to estimate animal dispersal, and this technique has also been applied to evaluate the connectivity of host populations in order to understand pathogen dispersal and spread [11–13]. Furthermore, understanding the relationship between populations and landscapes barriers among populations is essential for managing wildlife and reducing the outbreak of pathogens. Attributes of host species, such as their ecology, behavior, and dispersal ability, may be related to restricting pathogen dispersal or promoting it through dispersal corridors [11]. Therefore, understanding the population genetics and landscape barriers for wild boar would be useful to estimate CSFV spread and make strategies such as developing appropriate surveillance for the distribution of OMV and making barriers or centralized delivery of OMV around corridors of wild boar in order to prevent dispersal between populations.

In Japan, the quarantine area for OMV distribution was established by considering geographical characteristics (i.e., urban areas and alpine zones), and baits have been distributed by hand and by aircraft along and inside the quarantine area since 2019 under the Ministry of Agriculture, Forestry and Fisheries and each prefecture [14]. Oral bait vaccination is a key tool to prevent expansion of CSF, and therefore, it is important to deliver the oral bait vaccination effectively and efficiently [14].

Our previous study [15] conducted multiplexed inter-simple sequence repeat genotyping by sequencing (MIG-seq [16]) and clarified the genetic structure of wild boar in Fukushima prefecture in Japan [15]. That study indicated that (1) the genetic structure of wild boar is significantly different between groups inhabiting the East and West sides of the middle reaches of the Abukuma river and (2) the dispersal of wild boar may be affected by both the Abukuma river and urbanization along the river.

In this study, we hypothesized that if CSF tends to spread following the population structure of wild boar, the results of studying wild boar population structure could be useful (1) to predict the infection area of swine fever and (2) for selection of areas for vaccine application and management (e.g., concentrated application of the vaccine within and bordering wild boar populations where swine fever has occurred). Using wild boar populations in north-central Japan as a study system, our objectives were to clarify the geographic barriers that influence wild boar dispersal and to demonstrate the relationship between the spread of CSF infection within wild boars and their population structure.

## 2. Materials and Methods

### 2.1. Study Area

**Figure 1:**
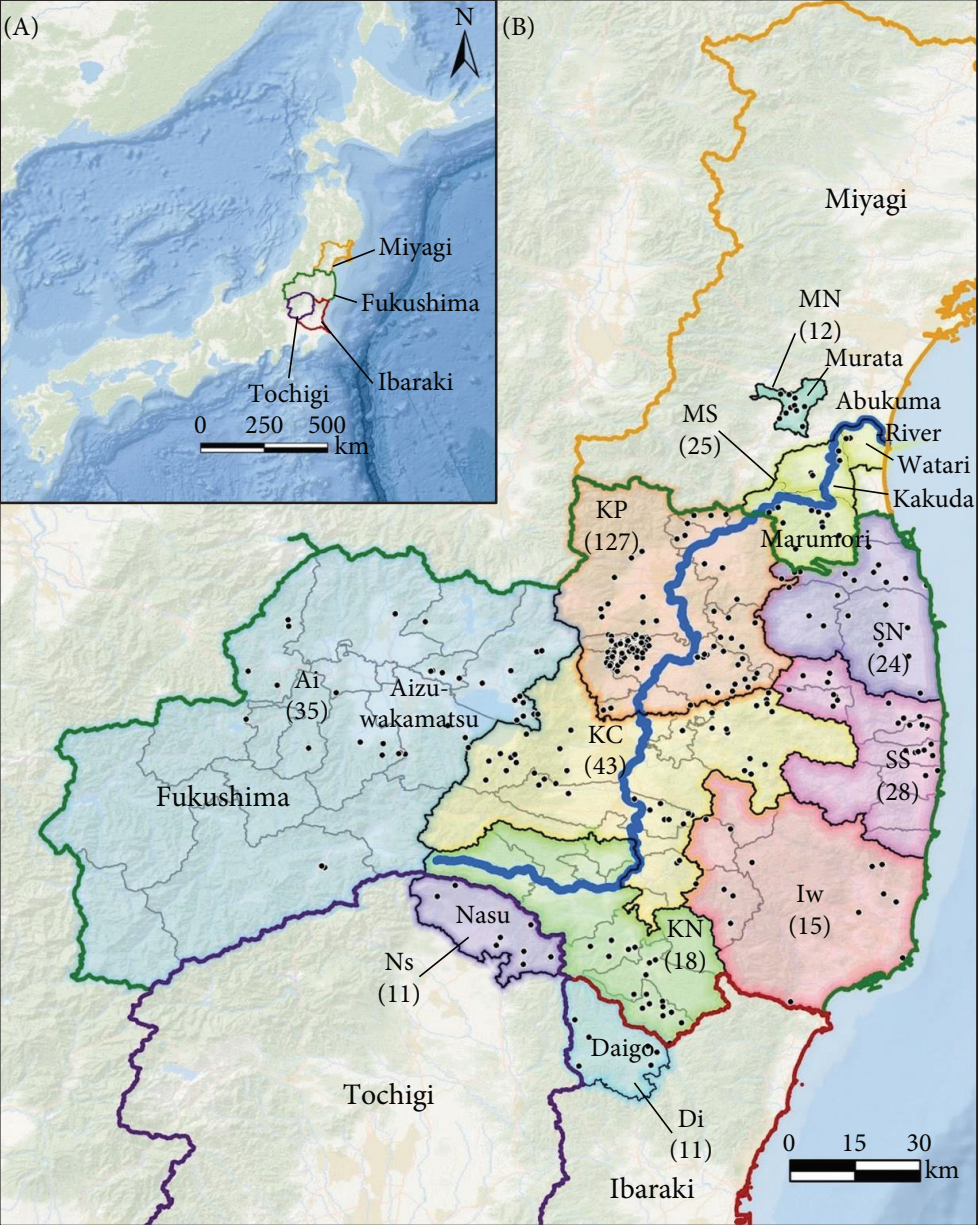
Sampling locations of wild boars. Locations include Fukushima, Miyagi, Tochigi, and Ibaraki prefectures (A). Administrative districts are delineated into 11 regions (B). Numbers in parentheses represent the number of wild boars analyzed in each district region. Sampling points of wild boars are shown by black dots. North of Soso (NS); South of Soso (SS); Iwaki (Iw); Ken-Poku (KP); Ken-Chu (KC); Ken-Nan (KN); Aizu (Ai); North of Miyagi (NM); South of Miyagi (SM); Nasu in Tochigi (Ns); and Daigo in Ibaraki (Di). Flow of Abukuma river is represented as a blue line.

We gathered wild boar tissue from a wide range of sites Fukushima prefecture, Ibaraki prefecture (Daigo town), Tochigi prefecture (Nasu town), and Miyagi prefecture (Kakuda city, Marumori town, Murata town, and Watari town) (Figure 1). Fukushima prefecture covers an area of 13,784 km^2^. It is characterized by two significant mountain ranges, the Abukuma Mountains and the Ou Mountains, situated on the east and west sides of the Abukuma river, respectively. These mountains result in climate differences between the east and west of Fukushima. Since April 2011, a part of Fukushima prefecture has been designated as an Evacuation Zone (hereafter, FEZ) due to radionuclide contamination resulting from the Fukushima Daiichi Nuclear Power Plant (hereafter, FDNPP) accident. There are no residents within the difficult-to-return zone (hereafter, DRZ) in the FEZ, and human presence on the landscape has been reduced. These environmental changes in the FEZ have contributed to increases in the population size of certain wildlife species, including wild boar [17]. Nasu town, located in the northern part of Tochigi prefecture and covers an area of 372 km^2^. Daigo town, covering 325 km^2^ in Ibaraki prefecture, is ~80% mountainous, consisting of the Yamizo and Abukuma mountain ranges. Miyagi prefecture covers an area of 7,282 km^2^. It is located on the east side of the Ou Mountains and faces the Pacific Ocean, and the climate in Miyagi is humid.

Based on distributional maps of wild boar produced by the Japanese Ministry of the Environment in 1978, 2003, 2011, 2014, and 2020 [18], wild boars have been present in east part of Fukushima, Daigo town in Ibaraki, east part of Nasu town in Tochigi, and south part of Marumori town in Miyagi since 1978. However, after 2011, the distribution of wild boar has expanded rapidly and wild boar now exists across a broad area in Fukushima, Ibaraki, Tochigi, and Miyagi prefectures, with the exception of northern Miyagi and southern Ibaraki [18].

### 2.2. Sample Collection

Since the Fukushima prefectural government gathered wild boar meat samples by hunters to measure the radioactive cesium concentration between 2013 and 2019 [19], we utilized these meat samples (i.e., thigh muscle). We also collected meat samples (thigh muscle) from the FEZ, including the DRZ and the area that was reopened to residents after having previously been evacuated. To avoid contamination, the surface was removed from the muscle block samples and minced. The sample that was used for measurement of radioactive cesium was stored in the refrigerator immediately after the measurement of radiocesium and then stored in freezer for long-term preserved. In addition, we collected wild boar tails captured in other three prefectures (i.e., Ibaraki, Tochigi, and Miyagi) in 2019 and 2020, then, took a sample from a piece of meat adhering to the tail. We collected wild boar meat samples (more than 2 g) from thigh muscle or tail.

In total, samples from 348 captured wild boar were used collected in Fukushima prefecture (*N* = 289), Ibaraki prefecture (*N* = 11), Tochigi prefecture (*N* = 11), and Miyagi prefecture (*N* = 37); Supporting Information Table S1, Figure 1B). Information on wild boar capture sites was obtained from maps or locations submitted by hunters (Figure 1B). Wild boar used in this study was caught by hunters as a part of efforts to control harmful wildlife implemented under the Wildlife Protection and Hunting Management Law (Law No. 32, 1918). In addition, wild boar meat captured in and around the FEZ was collected by the Ministry of the Environment. Therefore, wild boar was not killed specifically for this research. In addition, live animals were not used in this research.

These samples were stored in a freezer at −20 °C, or fixed in 99.9% ethanol, or freeze-dried and stored at room temperature until DNA extraction.

### 2.3. MIG-seq Analysis

DNeasy EZ1 Kit (QIAGEN, Germany) and BIO ROBOT EZ1 (QIAGEN) were used to extract genomic DNA. MIG-seq analysis was performed as previously described by Suyama and Matsuki [16] and Saito et al. [15, 20]. The first PCR was conducted using the following proportions with Multiplex PCR Assay Kit Ver.2 (Takara Bio, Kusatsu, Japan): 3.5 μL of 2× Multiplex PCR Buffer, 0.2 μM of each primer, and 0.035 μL of Multiplex PCR Enzyme Mix. Template DNA was added at 1.0 μL. The first PCR cycling conditions were as follows: 94 °C for 1 min, followed by 25 cycles of 94 °C for 30 s, 48 °C for 1 min, 72 °C for 1 min, with a final extension at 72 °C for 10 min. We conducted three times first PCR per sample. The success of first PCR amplification was confirmed by electrophoresis. Then, the three replicates of first PCR product were mixed and purified using AMPure XP (Beckman Coulter Life Sciences, San Jose, CA, USA). We used the purified PCR product as the template for the second PCR (Illumina, San Diego, CA, USA).

Each sample used different single index [16]. The second PCR was conducted using the following proportions with PrimeSTAR GXL buffer (Takara Bio, Kusatsu, Japan): 1.2 μL of 2.5 mM dNTP mixture, 3.0 μL of 5× PrimeSTAR GXL buffer, 0.375 U of PrimeSTAR GXL Polymerase, and primers at a final concentration of 0.2 μM each. Purified first PCR product was added at 3.0 μL. The second PCR cycling conditions were as follows: 98 °C for 10 s, 54 °C for 15 s, and 68 °C for 1 min for 12 cycles.

After the second PCR, PCR product was purified (QIAquick PCR Purification Kit, QIAGEN). To obtain 300–800 bp libraries, we performed size selection using SPRI Size Select (Beckman Coulter Life Sciences, San Jose, CA, USA). Then, the PCR products were separated on an agarose gel, and we cut out 300–800 bp fragments from the agarose gel. After that, the cut agarose gel piece purified using the QIAquick Gel Extraction Kit (QIAGEN). By Agilent 2200 TapeStation using the Genomic DNA ScreenTape System, we analyzed size distribution and concentration of the library. Sequencing was conducted by MiSeq (Illumina) using MiSeq Regent v3 150 cycle.

After the sequence, the first 14 bases of read 2 sequences were trimmed, then sequences of read 1 and trimmed read 2 were quality filtered (minimum quality score to keep (*q*) = 33, minimum percent of bases that must have “*q*” quality (*p*) = 40) using the FASTX-Toolkit ver.0.0.14 [21]. Then, TagDust v2.31 [22] was used to withdraw extremely short reads (i.e., containing primer sites in the sequence), and after that read 1 and trimmed read 2 data were combined. SNPs were extracted by Stacks v. 1.35 [23].

### 2.4. Data Analysis of MIG-seq Results

We performed STRUCTURE ver. 2.3.4 [24] to estimate genetic population structure using the obtained SNPs. First, to confirm the local genetic population structure roughly, wild boar samples were divided across 11 regional populations referring to administrative districts [i.e., seven regions in Fukushima prefecture, two regions in Miyagi, one region in Tochigi and Ibaraki, respectively (Figure 1B)], and then we conducted the STRUCTURE analysis. Based on our previous study [15], the first burn-in period was set to 100,000 times and performed 150,000 calculations by the Markov chain Monte Carlo (MCMC) method. We set the number of clusters (*K*) from 1 to 10, and 10 repetitions were conducted for each *K*. The Δ*K* [25] based on result of STRUCTURE was calculated using STRUCTURE Harvester ver. 0.6.94 [26]. The *K* value with the higher Δ*K* was regarded as the optimal number of clusters. After STRUCTURE analysis, we extracted data on the probability of membership to each clade for each wild boar via MIG-seq analysis based on STRUCTURE Harvester ver. 0.6.94. Hierarchical cluster analysis was performed via Ward's method using the default “stat” function implemented in the default R program [27], and then tree diagrams were created. We calculated the fixed index of genetic differentiation (*F*st) among the four groups (*K* = 2 and *K* = 4, following the results of hierarchical cluster analysis) using AMOVA approach in GenAlEx 6.503 [28]. The statistical significance of pair-wise *F*st values and AMOVA were assessed based on 9999 permutations with the “interpolate missing” option in GenAlEx 6.503. *p* < 0.05 of pairwise *F*st values were corrected for multiple comparisons using the Bonferroni method [29].

To predict the distributional range of each genetic population, we made predicted distribution maps in ArcGIS Pro using the data from the probability of each clade from our STRUCTURE analysis (*K* = 2 and *K* = 4). The predicted distribution map for each clade was made using an inverse distance weight (IDW) that interpolates a surface from points.

### 2.5. Genetic Difference Between East and West Populations of Abukuma River

To confirm whether the Abukuma river itself acts as a geographic barrier influencing the dispersal of wild boar populations, we compare the East and West populations of wild boar on the border of the Abukuma river in Fukushima and Miyagi prefectures. Then, hierarchical cluster analysis for wild boar regions [east-Fukushima (EF), west-Fukushima (WF), east-Miyagi (EM), and west-Miyagi (WM)] was performed via Ward's method using the default “stat” function implemented in the default R program [27].

### 2.6. Spatial Spread and Probability of CSF-Infected Wild Boar Belonging to Each of the Four Clades

**Figure 2:**
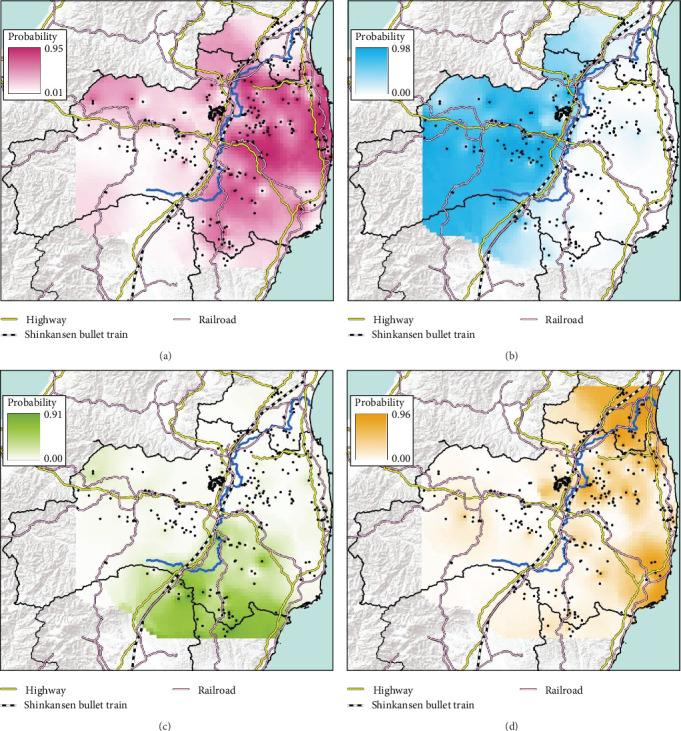
The map of the predicted distributional range of each clade of wild boar in Fukushima and its neighboring prefectures; East clade (A), West clade (B), South clade (C), and North clade (D) in STRUCTURE at *K* = 4. Each color gradation showed the probability of containing each clade. Blue line represents flow of Abukuma river.

**Figure 3:**
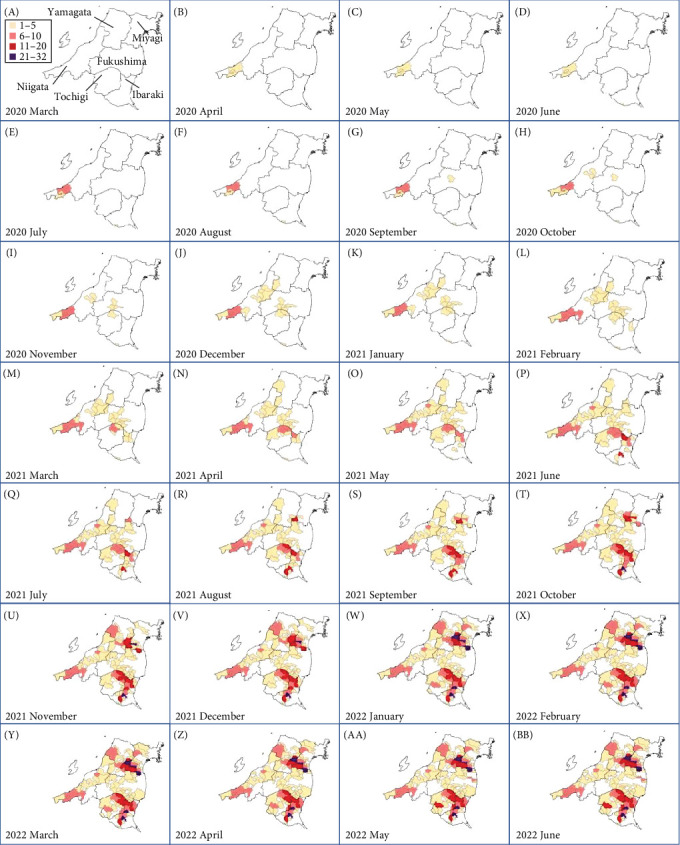
Temporal change of the total number of CSF-infected wild boar detection areas across the study area (Fukushima, Miyagi, Ibaraki, and Tochigi) and main neighboring prefectures of study area (Yamagata and Niigata) from March 2020 to June 2022. Each color represents a total number of CSF-infected wild boar in each municipality (refer to Supporting Information S6).

The conventional PCR method (i.e., CSFV-specific RT-qPCR) is mainly conducted to test for CSF in wild boar and is used in disease appraisal work in each prefecture [30]. All of the results of CSF-test for wild boar samples, whether positive or negative, are released on the website of each prefecture and the Japanese Ministry of Agriculture, Forestry, and Fisheries website [31–35]. We collected either the date that CSF tests were administered [32, 33] or the capture date and location of only CSF-infected wild boar [34, 35] in our study site from the website of each prefectural government [32–35]. We counted the total number of CSF-infected wild boar in each municipality and indicated the result of the cumulative number of positive boars on the map. To confirm the genetic traits of individual CSF-infected wild boar, we extracted the probability of containing each of the four clades (i.e., east, west, north, and south clades, *K* = 4) of CSF-infected wild boar belonging based on the predicted distribution map (i.e., Figures 2 and 3). However, we were unable to extract the six wild boar's sample data from five location (i.e., two samples were captured at the same location) because they were out of the range of the predicted distribution map (i.e., *K* = 4, Figure 2).

We used the capture location of wild boar as a target feature and used the extract multi values to points option based on the predicted distribution map of each clade using “Spatial joint” in ArcToolBox within ArcGIS Pro. In addition, we used representative location data in each municipality for Miyagi prefecture as the capture location was only recorded at the municipal level and detailed capture locations of CSF-infected wild boar were not available.

We obtained maps of each prefecture used in figures from the Ministry of Land, Infrastructure, Transport and Tourism (MLIT) of Japan [36]. ArcGIS Pro 3.1.6 (Esri, USA) was used to create the maps in figures.

## 3. Results

### 3.1. Estimation of Genetic Population Structure of Wild Boar

**Figure 4:**
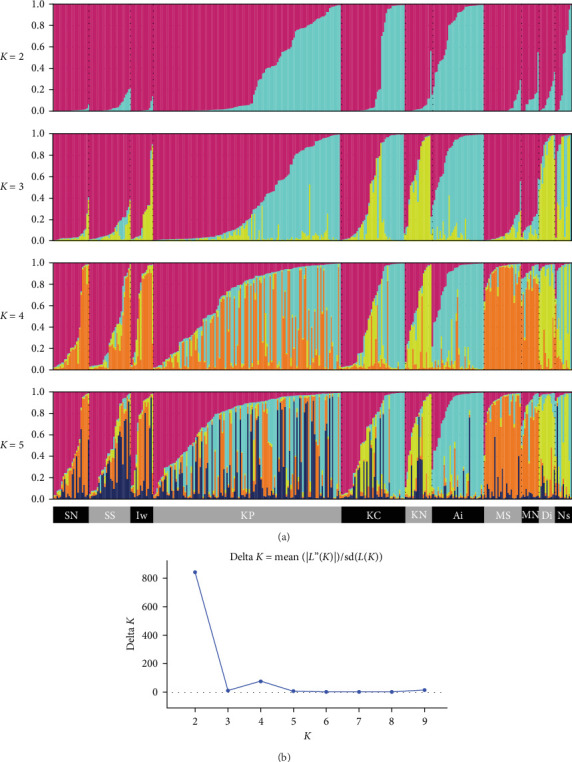
Results of the STRUCTURE analysis for *K* = 2–5 (A) and results of delta (Δ) *K* calculation based on STRUCTURE Harvester (B). The gray and black color bars indicate 11 regions (refer to Figure 1B). North of Soso (NS); South of Soso (SS); Iwaki (Iw); Ken-Poku (KP); Ken-Chu (KC); Ken-Nan (KN); Aizu (Ai); North of Miyagi (NM); South of Miyagi (SM); Nasu in Tochigi (Ns); Daigo in Ibaraki (Di).

**Figure 5:**
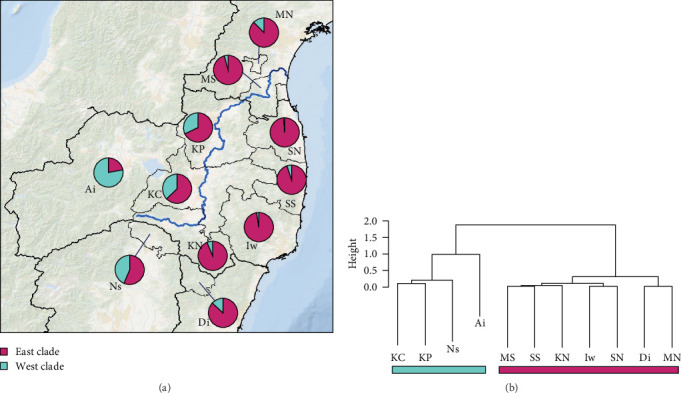
Rate of probability of belonging to each of the two clades (*K* = 2) in each region (A) and results of the cluster analysis for 11 regions of wild boars (B). Location of each region is provided in Figure 1B. Probability of belonging to each of the two clades was based on the results of STRUCTURE analysis (*K* = 2, Figure 4). Note: North of Soso (NS); South of Soso (SS); Iwaki (Iw); Ken-Poku (KP); Ken-Chu (KC); Ken-Nan (KN); Aizu (Ai); North of Miyagi (NM); South of Miyagi (SM); Nasu in Tochigi (Ns); Daigo in Ibaraki (Di). Flow of Abukuma river is represented as a blue line.

**Figure 6:**
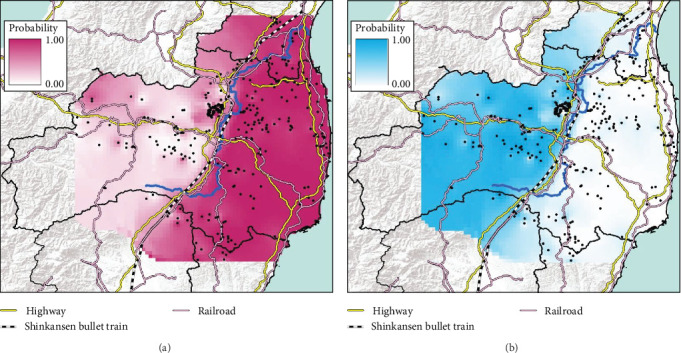
The map of the predicted distributional range of each clade of wild boar in Fukushima and its neighboring prefectures; East clade (A) and West clade (B) in STRUCTURE at *K* = 2. Each color gradation showed the probability of containing each clade. Blue line represents flow of Abukuma river.

We obtained 382 SNPs using MIG-seq analysis from wild boar samples collected in Fukushima and its three neighboring prefectures (Miyagi, Tochigi, and Ibaraki). The results of STRUCTURE analysis indicated that the highest Δ*K* value was at *K* = 2 (Δ*K* = 839.5), followed by *K* = 4 (Δ*K* = 74.7) (Figure 4B). Therefore, *K* = 2 is the most, and *K* = 4 is the second likely representation of the uppermost hierarchical level of genetic structure. Thus, we used results of *K* = 2 and *K* = 4 for subsequent analysis. The cluster analysis for *K* = 2 classified into two groups: (1) SN, SS, Iw, KN, MN, MS, and Di and (2) KP, KC, Ai, and Ns (Figure 5). In addition, the dendrogram and the prediction map for *K* = 2 showed two distinct clusters dominant in the regions of eastern Fukushima, Miyagi, Ibaraki, and eastern Tochigi (the dark pink cluster, i.e., East clade), and western Fukushima and western Tochigi (sky blue cluster, i.e., West clade) (Figures 5 and 6). The *F*st value among the two regions following the cluster analysis results of *K* = 2 was 0.039 (*p* < 0.001). The cluster analysis for *K* = 4 classified into four groups: (1) SN, SS, and Iw; (2) KC, KP, and Ai; (3) MN and MS; and (4) KN, Ns, and Di (Supporting Information Figure S2). Furthermore, the pie charts, dendrogram, and the prediction map for *K* = 4 showed four distinct clusters dominant in the regions of eastern Fukushima (the dark pink cluster, i.e., East clade), Miyagi (the orange cluster, i.e., North clade), southern Fukushima, Ibaraki, and eastern Tochigi (the light green cluster, i.e., South clade), and western Fukushima and western Tochigi (light blue clusters, i.e., West clade) (Figure 2, Supporting Information Figure S2). We calculated *F*st values among all four regions following the cluster analysis results of *K* = 4 (i.e., East region: SN, SS, and Iw population; North region: MN and MS population; South region: KN, Ns, and DI population; West region: KC, KP, and Ai population, refer to Figure 1, Supporting Information Figure S2), and the result showed that significant *F*st values ranged from 0.033 to 0.079 (*p* < 0.05, Supporting Information Table S3).

Compared to the results at *K* = 2, the results of *K* = 4 reflect more fine-scale genetic population structure in this area. The western clade at *K* = 4 exhibited a distribution trend similar to the western clade at *K* = 2. In contrast, the eastern clade at *K* = 2 was further divided into three subclades (i.e., East, North, and South clades) at *K* = 4.

### 3.2. The Abukuma River Does Not Act as a Geographic Barrier Influencing the Dispersal of Wild Boar

**Figure 7:**
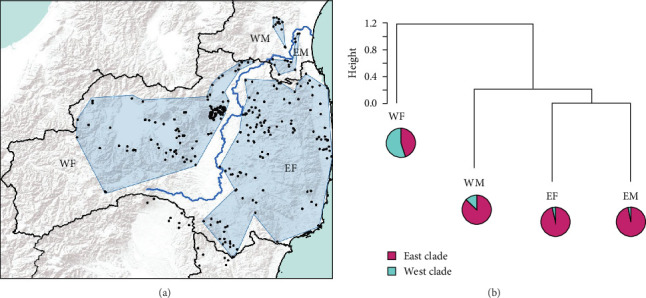
Four populations (east-Fukushima (EF), west-Fukushima (WF), east-Miyagi (EM), and west-Miyagi (WM)) (A) and results of the cluster analysis (B). Probability of belonging to each of the two clades was based on the results of STRUCTURE analysis (*K* = 2, Figure 4). Sampling points of wild boars are shown by black dots, and the enclosed lines indicate each population. Flow of Abukuma river is represented as a blue line.

The analysis of the classification of the four wild boar regions [east-Fukushima (EF), west-Fukushima (WF), east-Miyagi (EM), and west-Miyagi (WM), Figure 7] showed that EM and EF were classified closely together, while WM was slightly more distant from these two populations. The percentage of the West clade was slightly higher in WM than EM and EF. However, the East clade predominated in WM as well as EM and EF. On the other hand, WF predominantly belonged to the West clade and was distinct from the other three populations. Eventually, wild boar can be genetically classified into two groups: one including EF, EM, and WM and the other including only WF (Figure 7). Therefore, this indicates that the genetic population structure of the west-Miyagi region is not different from the east-Miyagi wild boar population.

### 3.3. Spatial Spread of the CSF-Infection Wild Boar

**Figure 8:**
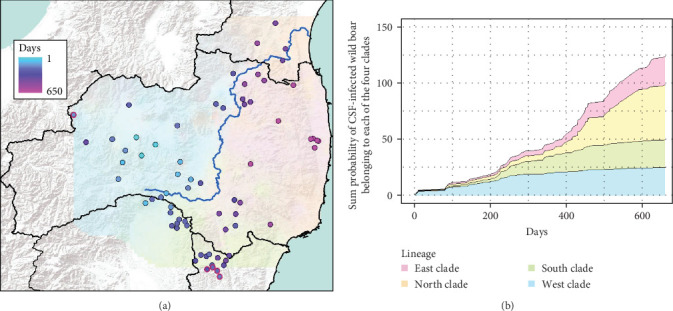
Temporal change of capture sites of CSF-infected wild boar (A) and sum of the probability of individual CSF-infected wild boar belonging to each of the four clades (B). Days indicate elapsed date after first CSF-infected wild boar was discovered in Aizuwakamastu city in Fukushima prefecture. Six samples (five locations) of individual CSF-infected wild boar that the probability of each genetic clade was unavailable were indicated with a red bold line circle. Flow of Abukuma river is represented as a blue line. The background colors on the map indicate the results of the predicted distributional range of the East clade (dark pink), West clade (sky blue), South clade (light green), and North clade (orange) by Bayesian model-based clustering in STRUCTURE at *K* = 4.

Fukushima, Miyagi, Tochigi, and Ibaraki prefectures all began testing from September 2018. The first positive individuals were confirmed in Fukushima prefecture in September 2020, Miyagi prefecture in June 2021, Tochigi prefecture in November 2020, and Ibaraki prefecture in June 2020. The number of CSF-infected wild boar has increased over time since its introduction (Supporting Information Figure S4–S6). Based on our results, the spread of CSF-infected wild boar in Fukushima and its neighboring prefectures appears to reflect the underlying wild boar genetic population structure within this region (Figures 2 and 3, Supporting Information S6). In September 2020, the first occurrence of CSF in Fukushima prefecture was confirmed in Aizuwakamatsu city, located in the western area of Fukushima prefecture (Figure 3A–G). After that, CSF spread southward to other areas in western Fukushima and Tochigi prefectures (Figure 3G–K), corresponding with the distributional area of the West clade wild boar population. In February 2021, CSF was confirmed in eastern Tochigi, the northern part of Ibaraki, and the southern area of Fukushima prefecture (i.e., the range of South clade of wild boar, Figures 2 and 3L–N). Almost at the same time, CSF was reported throughout the western area of Fukushima prefecture and extended northward, with the first occurrence of CSF confirmed in Miyagi prefecture in June 2021 (Figure 3O and P), indicating CSF spread into North clade wild boar (Figures 2 and 3O, P). After CSF infections spread within the southern area of Miyagi prefecture, the first occurrence of CSF in north-eastern Fukushima prefecture was confirmed (Figures 2 and 3W). In addition, CSF infections in wild boar in south-eastern Fukushima occurred gradually (Figures 2 and 3V–Z). The first case of CSF inside the Fukushima evacuation zone was confirmed after May 2022 (Figure 3AA and BB). The time-series change in (1) the capture site of CSF-infected wild boar and (2) the sum of the probability of belonging to each of the four clades in individual CSF-infected wild boar (Figure 8), clearly indicated that the majority of CSF-infected wild boar initially had a high probability of belonging to the West clade. After that, the rate of the West clade of CSF-infected wild boar increased over time. In addition, the probability of belonging to the South clade of CSF-infected wild boar also increased by 218 days after detection. By February 2022 (around 520 days after detection), the spread of CSF in wild boar followed the sequence: West clade, South clade, North clade, and finally East clade (Figure 8). They also indicated that the spread of CSF to the eastern part of Fukushima prefecture likely occurred through the northern wild boar population rather than the western population (Figure 8).

## 4. Discussion

Our results clearly indicated that wild boar exhibit genetic differences within a relatively small area but that the Abukuma river does not appear to be a barrier to the gene flow. In addition, the genetic structure of wild boar in our study area was correlated with the outbreak of CSF, suggesting population genetics can be useful for predicting transmission of diseases within a region. Predictions of disease spread, especially for widely distributed host species, are challenging because of the risk of cryptic breaks and changes in wide range connectivity; however, genetic analysis of host population structures may hold the possibility to improve future predictions of disease spread [13]. Given the global distribution of wild boar and concerns over the spread of CSFV and other transboundary diseases, further investigation of the genetic population structure of this species across their range should be prioritized to better understand potential transmission pathways.

Our results showed that wild boar can be classified into distinct genetic population structures over relatively small geographic regions. Further, by including additional samples, particularly from neighboring prefectures of Fukushima (i.e., Miyagi, Ibaraki, and Tochigi) as well as the southern area of Fukushima prefecture (e.g., Iw and KN, Figure 1), our study provided a more detailed and fine-scale understanding of the genetic population structure of wild boar compared to our previous study [15]. Notably, a clear geographical distribution trend was observed in within the geographic extent of our sampling, with distinct East and West clades (Figures 5 and 6). In Japan, the Ministry of the Environment created a distributional map of wild boar in 5 km mesh units, based on surveys conducted in FY1978, 2003, 2011, and 2014. This map incorporates information on capture locations obtained from capture permits and interviews with prefectures [18]. When comparing the wild boar distributional map with our findings on the genetic population structure of wild boar (*K* = 2), we observed that the East clade in our results corresponds to the distribution of wild boar in 1978 and 2003. On the other hand, the West lineage was primarily detected in the western part of Fukushima and Tochigi prefectures, aligning with areas where their distribution has expanded in recent years (i.e., since 2011). Therefore, the West clade of wild boar succussed to be expanding their distribution range where wild boar was not distributed before. Thus, comparison between the results of the wild boar distribution surveys and the genetic population structure would be useful to identify the genetic origins of these expanding populations.

Wild boar (and wild pigs) is widely distributed globally, and their population size has been increasing across their range over the past few decades [37–39]. Although the distributional range of wild boar is limited by factors such as temperature, availability of water, depth of snow, vegetation cover, and large carnivore richness (e.g., Lewis et al. [37]; McClure et al. [38]), understanding the mechanisms contributing to the recent expansion of wild pig populations is a priority area of research. Within Japan, the underlying factors contributing to the expansion of wild boar populations are still unclear; however, environmental change resulting from shifts in human-life style, such as decline in forest utilization and cultivated land, has likely increased the availability of habitats for wild boar [40]. Within our study region, where wild boar populations have recently expanded, it is possible that the environment in the western area has undergone changes due to climate change, such as alterations in temperature and snow accumulation, allowing wild boar to disperse from neighboring areas and expand their distribution.

Previous studies have suggested rivers can serve as barriers to species distribution or the gene flow between populations (e.g., riverine barrier hypothesis) (e.g., some non-volant small mammals [41, 42]; arboreal primates and [43, 44]; wild boar [45]), however, other studies have indicated that rivers are not absolute barriers to gene flow (e.g., wild boar [46, 47]). Therefore, whether a river acts as a barrier probably depends on the species and the size of the river. In our study, it is evident that the Abukuma river itself does not act as a barrier to the gene flow of wild boar. Wild boars are expected they are able across rivers, lakes, and ocean because of their swimming ability [48, 49]. Therefore, Abukuma river is not absolute barriers to gene flow for wild boars. However, human infrastructure, such as highways and railways, appears to hinder the dispersal of wild boar [50]. This is evidenced by the fact that the genetic differences observed between the wild boar groups inhabiting the eastern and western areas across the Abukuma river are only found in Fukushima prefecture (Supporting Information Figure S7). Furthermore, the South clade population (*K* = 4) is distributed in a specific area that is surrounded by highways, which may act as boundaries that restrict gene flow.

In comparison to the results of *K* = 2 (i.e., East and West clades), the boundaries and barriers of three clades of *K* = 4 (i.e., East, North, and South clades) are not clearly defined. This suggests that either the geographical barriers preventing gene flow between eastern and western population are strong, there is a moderate connectivity between three clades of *K* = 4 (i.e., East, North, and South clades), or genetic differentiation among these three populations has occurred in recent years. Further study is required to comprehend the factors that impeded gene flow in the observed subpopulations (East, North, and South) at *K* = 4. In northern Fukushima (approximately the boundary area between the eastern and northern wild boar population), a new highway spanning ~45 km was opened in 2021. In addition, there have been land use changes in Japan, including within our study area, such as the decline and depopulation of rural areas. In the future, these changes in anthropogenic influence may impact the dispersal range of wild boars and their population structure.

In the results of spread of CSF-infected wild boar, despite the close proximity between eastern and western parts of Fukushima, CSF-infected wild boar was found at a faster and higher rate in Miyagi prefecture compared to eastern Fukushima prefecture after the initial CSF-infected wild boar were detected in the western part of Fukushima prefecture. These results coincide with the results of a genetic difference between eastern and western wild boar populations, attributed to human infrastructure influencing their dispersal. Therefore, the genetic population of wild boar might be useful in predicting the outbreak areas of CSF and estimating disease dynamics. Shimizu et al. [2] mentioned that disease surveillance in wild boar is an essential task due to their important role in the spread of the CSF.

According to Shimizu et al. [13], CSF spread faster to the north and east, which is dominated by forested land, than to the south and west, which contains more urban areas after September in 2018. Based on our finding that a correlation exists between the spread of CSF during the outbreak and wild boar genetic structure, we suggest that future CSF monitoring and mitigation efforts would benefit from a clearer understanding of wild boar population genetics. This method can aid in early detection of outbreaks and provide insight into infection dynamics. Similar to our study, Reiner et al. [51] investigated the genetic population structure of wild boar in the Rhineland-Palatinate area of Germany using microsatellite markers and estimated the possible barriers to their movements to develop strategies for combating the spread of African Swine Fever (ASF). They suggested fencing and intensive hunting following the identified barriers of wild boar dispersal before an ASF outbreak as potential management strategies for controlling an outbreak of ASF [51]. In addition, we suggest that the understanding of genetic population structure may be used to develop a vaccination strategy that considers wild boar population structure and would be effective in reducing the spread of infection by ensuring efficient delivery of oral vaccinations. Indeed, in addition to wild boar, oral vaccination has been successfully implemented for controlling other diseases affecting wildlife hosts, such as rabies (e.g., Dixon et al. [52], Elmore et al. [53]).

In addition to CSF, wild boars are also susceptible to other transboundary diseases such as African Swine Fever virus (ASFv). According to a study employing a spatially explicit disease transmission model of ASFv that contrasts wild pig movement and contact ecology, wild pig movement was identified as a more reliable predictor of the optimal response area than the number of ASFv cases early in the outbreak trajectory [54]. To prepare for these threats, understanding the dispersal and genetic structure of wild boar populations across their range is needed to help to optimize disease control and allow management efforts such as identify the outbreak routes [51, 55]. Thus, population genetics may be an effective tool for understanding and managing wildlife diseases that span large spatial extents.

## Data Availability

Accession codes: Raw MIG-seq data are deposited at the DDBJ Sequence Read Archive (DRA) with accession number; DRA012666 and DRA013630 (Submission), PRJDB12172 and PRJDB13082 (BioProject), SAMD00399887–SAMD00400078 and SAMD00443740–SAMD00443914 (BioSample), and DRR316310–DRR316501 and DRR352100–DRR352274 (Run). The data that support the findings of this study are presented in the main figures and supporting information of this article. Further information and requests should be directed to the lead contact. Data will be made available on request.

## Supporting Information

Supporting Information 1 Supporting Information. Table S1: Sample ID, species name, captured site (prefecture and City/Town/Villege), location distriction in Fukushima, Miyagi, Tochigi, and Ibaraki prefectures (refer to Figure 1), location of East/West side of Abukuma river, and DRX Number of DDBJ. Supporting Information. Table S3: Pairwise *Fst* value (below diagonal) and *p* value (above diagonal) among the clade in Fukushima, Miyagi, Tochigi, and Ibaraki prefectures based on 382 SNPs data (*K* = 4) calculated using an AMOVA approach.

Supporting Information 2 Supporting Information. Figure S2: Rate of probability of belonging to each four clades (*K* = 4) in each region (A) and results of the cluster analysis for 11 regions of wild boars (B). Location of each region is provided in Figure 1B. Probability of belonging to each of the two clades was based on the results of STRUCTURE analysis (*K* = 4, Figure 4). North of Soso (NS); South of Soso (SS); Iwaki (Iw); Ken-Poku (KP); Ken-Chu (KC); Ken-Nan (KN); Aizu (Ai); North of Miyagi (NM), South of Miyagi (SM); Nasu in Tochigi (Ns); Daigo in Ibaraki (Di). Flow of Abukuma river is represented as a blue line. Supporting Information. Figure S4: Monthly temporal change of the results of CSF-infected test of wild boar across the study area from September 2020 to June 2022. Dot circle indicates the capture site of wild boar. Each color represents a result of CSF-test (Red: Positive, and Blue: Negative). Flow of Abukuma river is represented as a blue line. The results of CSF-infected test is based on Ministry of Agriculture, Forestry and Fisheries in Japan: https://www.maff.go.jp/j/syouan/douei/csf/wildboar_map.html (accessed December 5, 2023). Supporting Information. Figure S5: Annual number and results of the CSF-infected test of wild boar in Miyagi, Fukushima, Tochigi, and Ibaraki prefectures from September 2018 to August 2022. M: Miyagi, F: Fukushima, T: Tochigi, and I: Ibaraki. The data are based on: Fukushima prefecture 2023, Miyagi prefecture 2023, Ibaraki prefecture 2023, Tochigi prefecture 2023. Supporting Information. Figure S7: Geographical features and land use around Fukushima Prefecture and its neighboring prefectures. The circular radiation symbol indicates the location of the Fukushima Daiichi Nuclear Power Plant. Flow of Abukuma river is represented as a blue line. The data are based on the MLIT of Japan: https://nlftp.mlit.go.jp/ksj/index.html (accessed December 5, 2023).

Supporting Information 3 Supporting Information S6: Temporal change of the total number of CSF-infected wild boar detection areas across in each municipality of six prefectures (Fukushima, Miyagi, Ibaraki, Tochigi, Yamagata, and Niigata) from March 2020 to June 2022.
