# Supplementary material for: Exploring the Genetic Basis of Wild Boar (Sus scrofa) and Its Connection to Classical Swine Fever Spread
Source: Transbound Emerg Dis. 2025 May 7;2025:9881511. doi: 10.1155/tbed/9881511 (PMC12077976; doi:10.1155/tbed/9881511)
Supplement: Supporting Information 2 — Supporting Information. Figure S2: Rate of probability of belonging to each four clades (K = 4) in each region (A) and results of the cluster analysis for 11 regions of wild boars (B). Location of each region is provided in Figure 1B. Probability of belonging to each of the two clades was based on the results of STRUCTURE analysis (K = 4, Figure 4). North of Soso (NS); South of Soso (SS); Iwaki (Iw); Ken-Poku (KP); Ken-Chu (KC); Ken-Nan (KN); Aizu (Ai); North of Miyagi (NM), South of Miyagi (SM); Nasu in Tochigi (Ns); Daigo in Ibaraki (Di). Flow of Abukuma river is represented as a blue line. Supporting Information. Figure S4: Monthly temporal change of the results of CSF-infected test of wild boar across the study area from September 2020 to June 2022. Dot circle indicates the capture site of wild boar. Each color represents a result of CSF-test (Red: Positive, and Blue: Negative). Flow of Abukuma river is represented as a blue line. The results of CSF-infected test is based on Ministry of Agriculture, Forestry and Fisheries in Japan: https://www.maff.go.jp/j/syouan/douei/csf/wildboar_map.html (accessed December 5, 2023). Supporting Information. Figure S5: Annual number and results of the CSF-infected test of wild boar in Miyagi, Fukushima, Tochigi, and Ibaraki prefectures from September 2018 to August 2022. M: Miyagi, F: Fukushima, T: Tochigi, and I: Ibaraki. The data are based on: Fukushima prefecture 2023, Miyagi prefecture 2023, Ibaraki prefecture 2023, Tochigi prefecture 2023. Supporting Information. Figure S7: Geographical features and land use around Fukushima Prefecture and its neighboring prefectures. The circular radiation symbol indicates the location of the Fukushima Daiichi Nuclear Power Plant. Flow of Abukuma river is represented as a blue line. The data are based on the MLIT of Japan: https://nlftp.mlit.go.jp/ksj/index.html (accessed December 5, 2023). [file 9881511.f2.docx]

**Supplementary Information**

**Title:** Estimating the spread of classical swine fever infection in wild boar (*Sus Scrofa*) using genetic population structure

Authors: Rie Saito ^1,2,3^*, Natsuko Ito Kondo ^4^, Yui Nemoto ^5^, Toshimasa Takeda^3^, Kosuke Kanda ^2^, Nobuyoshi Nakajima ^4^, James C. Beasley ^1,6^ Masanori Tamaoki ^3,4^

^1^ Savannah River Ecology Laboratory, University of Georgia, P O Drawer E, Aiken, SC, 29802, USA

^2^ Fukushima Prefectural Centre for Environmental Creation, 10-2 Fukasaku, Miharu-machi 963-7700

^3^ Fukushima Regional Collaborative Research Center, National Institute for Environmental Studies, 10-2 Fukasaku, Miharu-machi 963-7700, Japan

^4^ Biodiversity Division, National Institute for Environmental Studies, 16-2 Onogawa, Tsukuba, 305-8506, Japan

^5^ Okutama Practice Forest, Tokyo University of Agriculture, Hikawa 2137, Okutama 198-0212, Japan

^6^ Warnell School of Forestry and Natural Resources, University of Georgia, P O Drawer E, Aiken, SC, 29802, USA


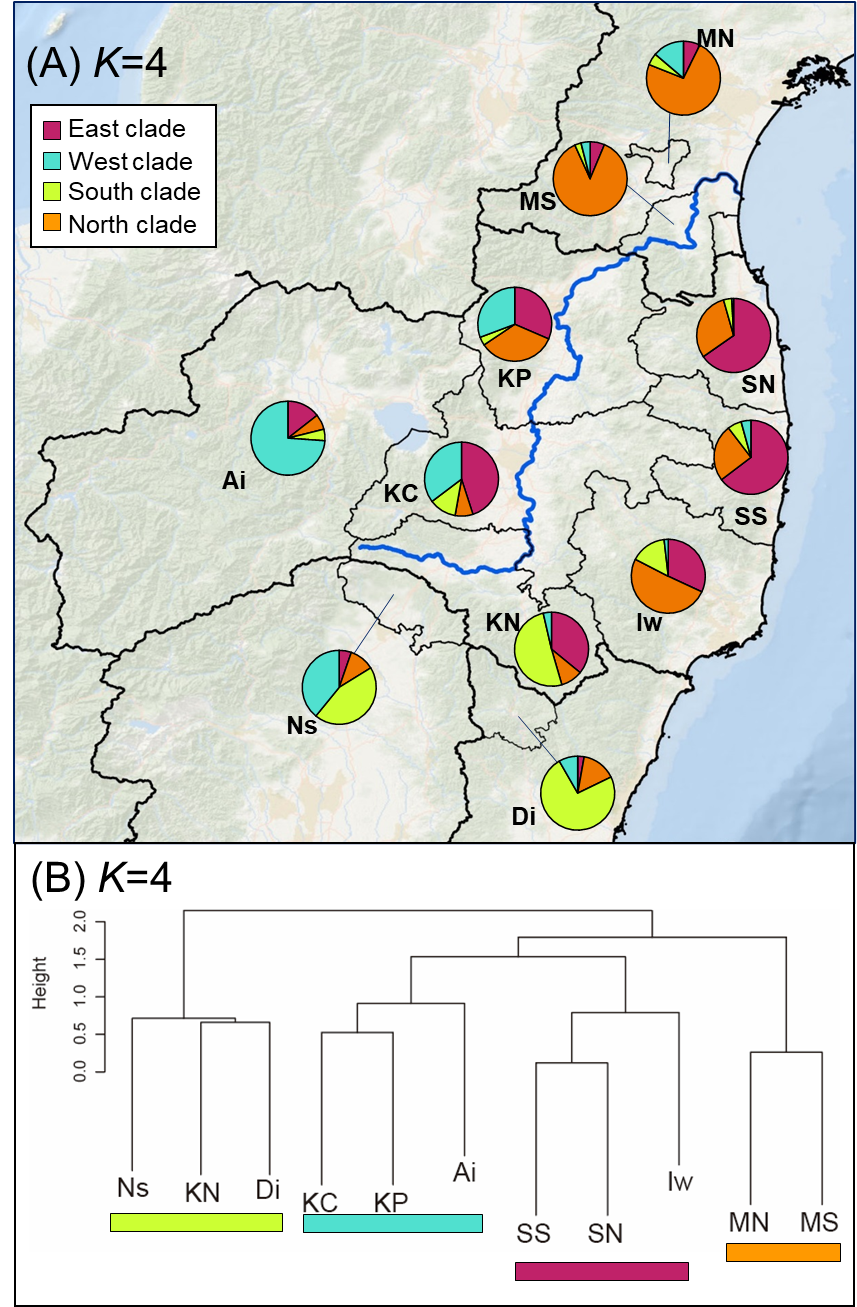
Supplemental 2 Rate of probability of belonging to each four clades (*K* = 4) in each region (A) and results of the cluster analysis for eleven regions of wild boars (B). Location of each region is provided in Figure 1B. Probability of belonging to each of the two clades was based on the results of STRUCTURE analysis (*K* = 4, Figure 2). North of Soso (NS); South of Soso (SS); Iwaki (Iw); Ken-Poku (KP); Ken-Chu (KC); Ken-Nan (KN); Aizu (Ai); North of Miyagi (NM), South of Miyagi (SM); Nasu in Tochigi (Ns); Daigo in Ibaraki (Di). Flow of Abukuma river is represented as a blue line.


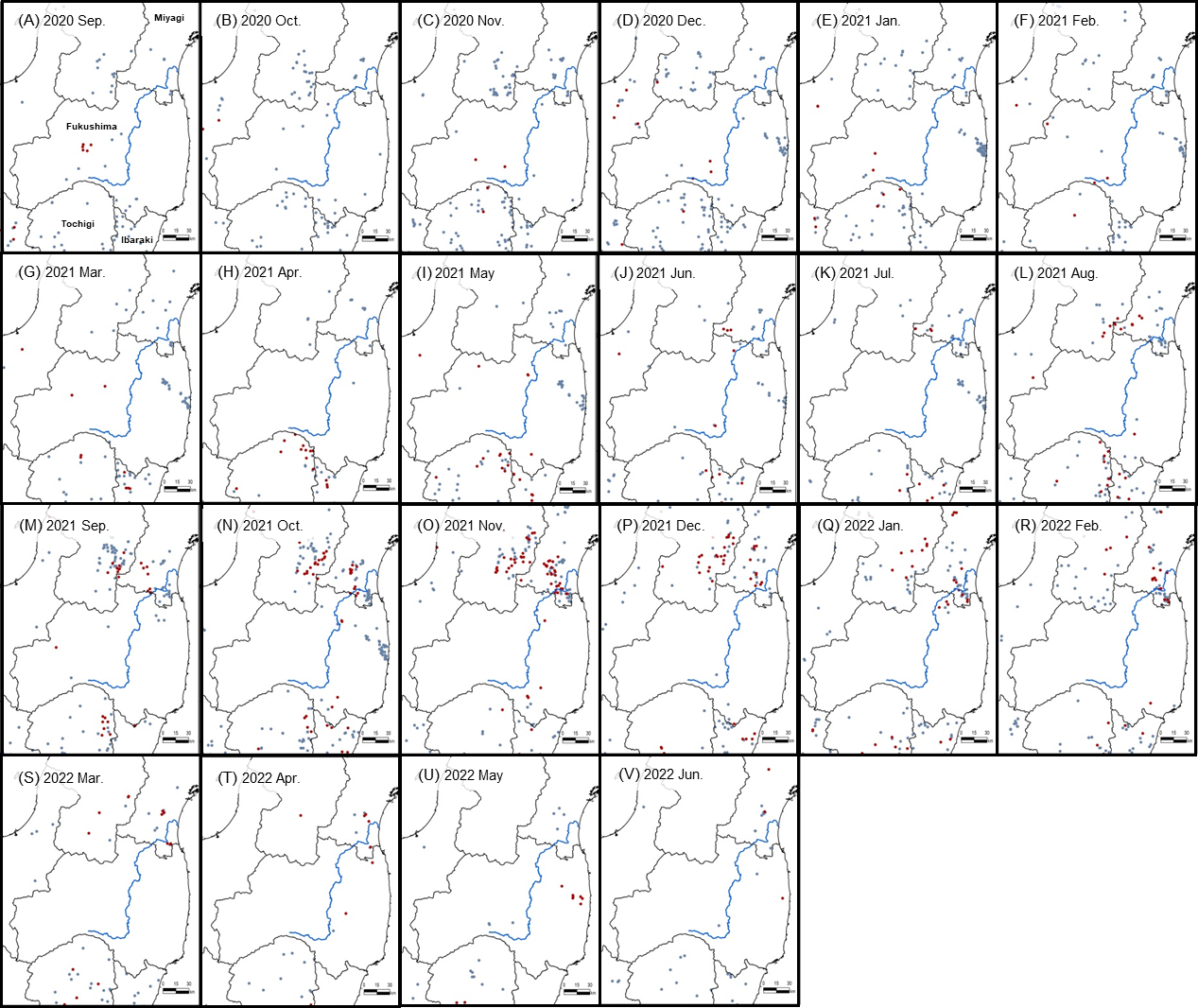


Supplemental Figure 4 Monthly temporal change of the results of CSF-infected test of wild boar across the study area from September 2020 to June 2022. Dot circle indicates the capture site of wild boar. Each color represents a result of CSF-test (Red: Positive, and Blue: Negative). The results of CSF-infected test is based on Ministry of Agriculture, Forestry and Fisheries in Japan: <https://www.maff.go.jp/j/syouan/douei/csf/wildboar_map.html> (accessed 05 December 2023).


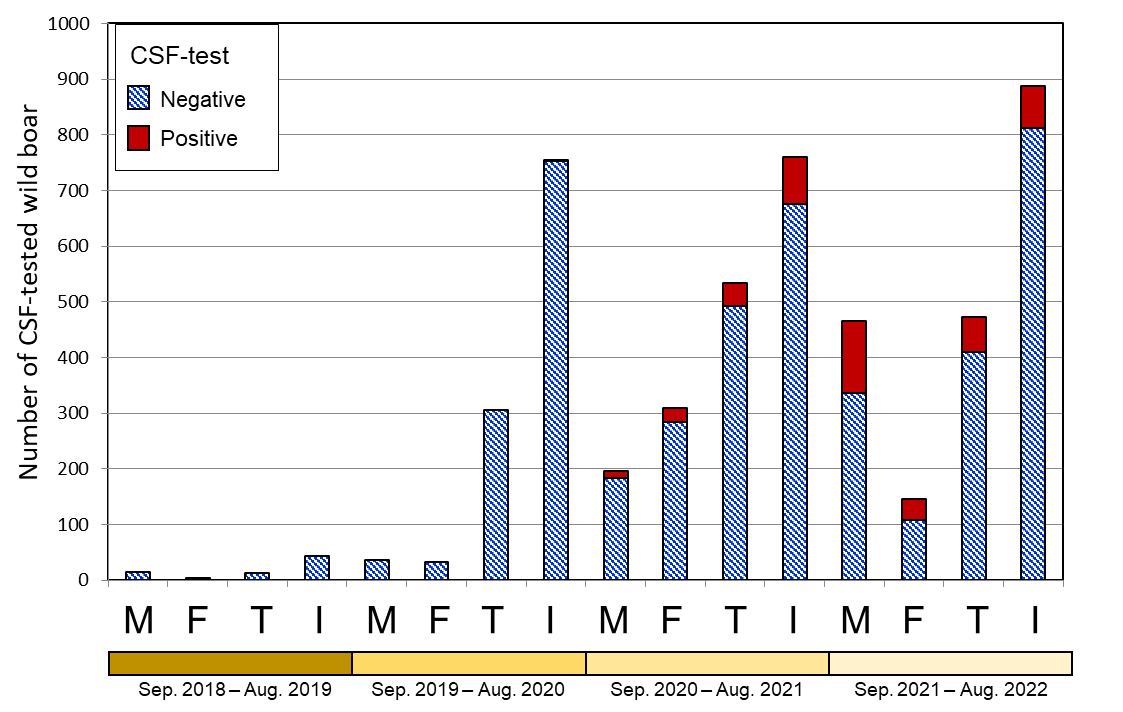
Supplemental 5. Annual number and results of the CSF-infected test of wild boar in Miyagi, Fukushima, Tochigi, and Ibaraki prefectures from September 2018 to August 2022. M: Miyagi, F: Fukushima, T: Tochigi, and I: Ibaraki. The data is based on: Fukushima prefecture 2023, Miyagi prefecture 2023, Ibaraki prefecture 2023, Tochigi prefecture 2023.


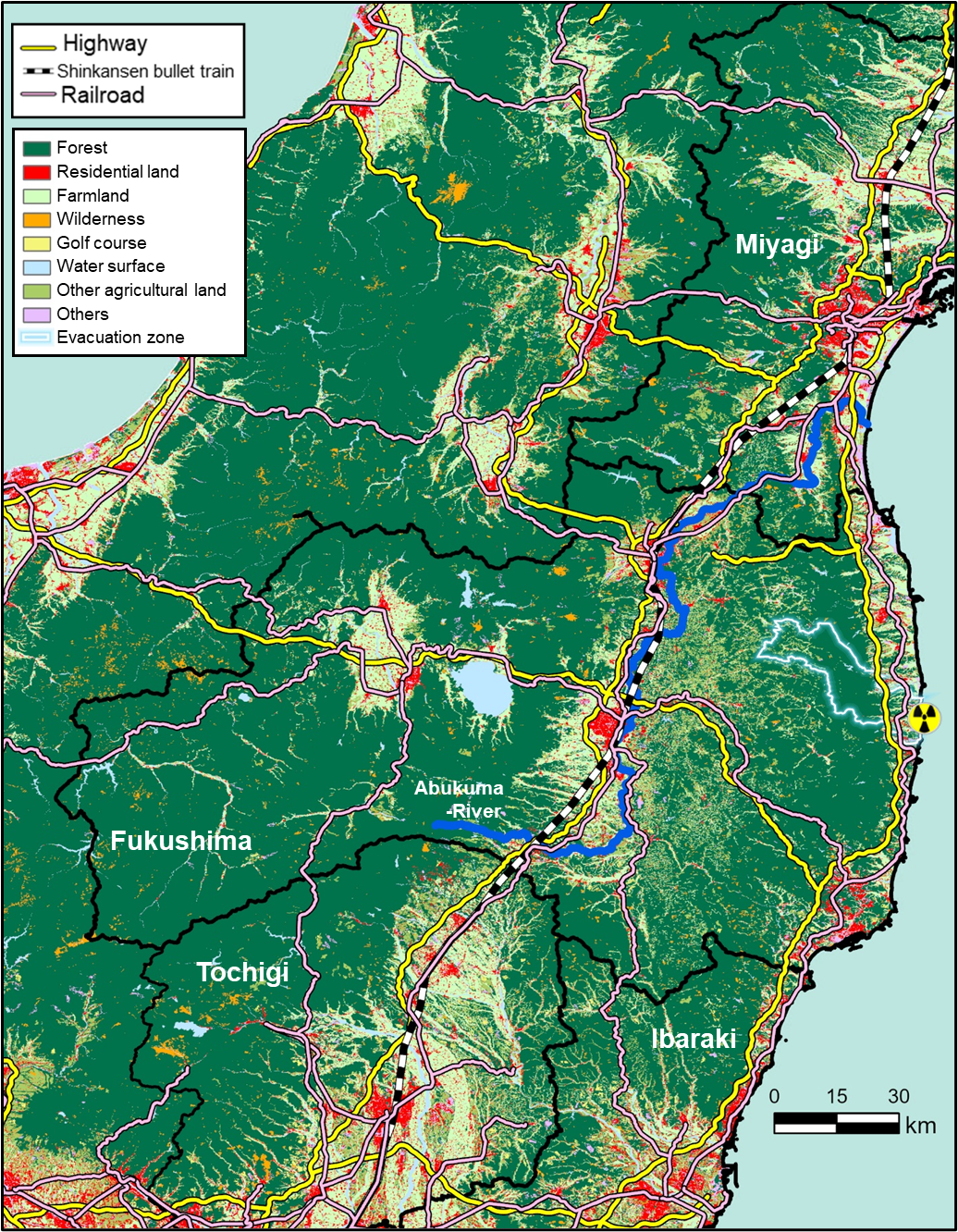


Supplemental 7. Geographical features and land use around Fukushima Prefecture and its neighboring prefectures. The circular radiation symbol indicates the location of the Fukushima Daiichi Nuclear Power Plant. The data is based on the MLIT of Japan: https://nlftp.mlit.go.jp/ksj/index.html (accessed 05 December 2023).
